# Gut Microbiome‐Targeted Preventive Strategies for Polyendocrine Metabolic Ovarian Syndrome: Insights From the Prenatal and Early‐Life Environment

**DOI:** 10.1002/rmb2.70097

**Published:** 2026-09-09

**Authors:** Akari Kusamoto, Yutaka Osuga, Yasushi Hirota, Miyuki Harada

**Affiliations:** ^1^ Department of Obstetrics and Gynecology, Faculty of Medicine University of Tokyo Tokyo Japan; ^2^ Teikyo Academic Research Center Teikyo University Tokyo Japan

**Keywords:** gut microbiome, intrauterine environment, polycystic ovary syndrome (PCOS), polyendocrine metabolic ovarian syndrome (PMOS), prenatal androgen exposure

## Abstract

**Background:**

Polyendocrine metabolic ovarian syndrome (PMOS), the proposed new name for polycystic ovary syndrome (PCOS), is a complex endocrine and metabolic disorder influenced by genetic and environmental factors. Despite its high prevalence and familial aggregation, no effective preventive strategy is available. Prenatal androgen exposure is considered a key factor in PMOS pathogenesis, with studies indicating a significant role for the gut microbiome.

**Methods:**

This review summarizes evidence from human and animal studies exploring the association between PMOS and the gut microbiome, spotlighting the impact of the intrauterine environment, including prenatal androgen exposure and maternal gut microbiome, in PMOS development.

**Main Findings:**

Gut microbiota dysbiosis is associated with features of PMOS pathophysiology, including hyperandrogenism, insulin resistance, neuroendocrine dysfunction, and ovarian dysfunction. Emerging evidence suggests that prenatal and early postnatal environments shape gut microbiota establishment and maturation, which may interact with multiple developmental pathways and potentially modify susceptibility to PMOS later in life.

**Conclusion:**

The gut microbiome may be involved in the developmental origins and pathophysiology of PMOS, although its precise role remains unclear. Understanding early‐life host‐microbiome interaction may provide a foundation for future preventive and personalized management strategies for PMOS, but further mechanistic studies and clinical validation are required.

## Graphical abstract

Maternal and early‐life factors shape gut microbiota development during critical developmental windows, with potential long‐term effects on metabolic and endocrine health. The gut microbiome may modify developmental programming and PMOS pathophysiology, supporting further investigation of early‐life microbiome‐targeted strategies.

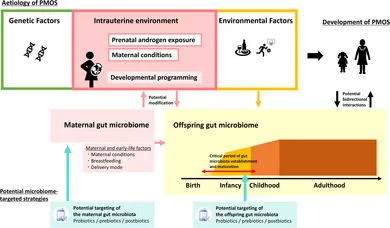

## 1 Introduction

Polyendocrine metabolic ovarian syndrome (PMOS), formerly known as polycystic ovary syndrome (PCOS), is a common, multifactorial endocrine and metabolic disorder among reproductive‐age women, with a prevalence of 10%–13%. Alongside reproductive abnormalities (e.g., hyperandrogenism [HA], ovulatory dysfunction [OD], and polycystic ovarian morphology [PCOM]), metabolic (e.g., insulin resistance, obesity, and dyslipidemia) and psychological comorbidities are frequently observed in PMOS. Reflecting its marked clinical heterogeneity and the recognition that PCOM is neither essential nor specific for diagnosis, the renaming of PCOS to PMOS has recently been proposed to better represent the syndrome's underlying pathophysiology [1]. Owing to the complex interactions and high heterogeneity of these characteristics, PMOS pathophysiology remains partially understood [2]. Moreover, PMOS demonstrates strong familial aggregation and involves a complex interplay of genetic susceptibility and environmental factors, including the prenatal intrauterine environment and postnatal lifestyle. However, currently identified genetic variants explain only a miniscule proportion of the estimated heritability, indicating that environmental factors considerably influence PMOS pathophysiology [3].

The gut microbiome has emerged as an important regulator of host metabolism, endocrine function, immune response, neuroendocrine signaling, and epigenetic processes, and has been implicated in the pathogenesis of various diseases [4]. Although in nascent stages, accumulating evidence suggests the involvement of the gut microbiome in PMOS pathogenesis. Recent studies have demonstrated alterations in the gut microbiota of both women with PMOS and animal models compared with their respective controls. Although the causal relationship remains under debate, findings from animal experiments using fecal microbiota transplantation (FMT) and other mechanistic investigations suggest that gut microbiota alterations may not simply represent a consequence of endocrine and metabolic abnormalities but could also influence disease development and progression through bidirectional interactions.

Early life, particularly the prenatal and early postnatal periods, represents a critical “window of opportunity” for gut microbiota establishment and maturation [5, 6]. Growing evidence suggests that maternal factors during pregnancy, including the maternal gut microbiota as well as metabolic and hormonal conditions, may influence both the establishment of the offspring gut microbiota and developmental programming, with potential long‐term effects on metabolic and reproductive health. Collectively, these findings suggest that the gut microbiome may act as a potential modifier linking maternal environmental factors with susceptibility to PMOS, although the precise causal relationships remain to be established.

In this review, we spotlight the intrauterine and early postnatal environments and summarize current evidence linking PMOS and the gut microbiome. We discuss proposed mechanisms, insights from animal and human studies, and the potential role of the microbiome as a modifier of disease susceptibility. Finally, we highlight future research directions and discuss the potential implications of microbiome‐based approaches for the prevention and management of PMOS.

## 2 Polyendocrine Metabolic Ovarian Syndrome

### 2.1 Clinico‐Biological Features of PMOS

The Rotterdam criteria for PMOS diagnosis require the presence of at least two of the following three symptoms: (i) clinical and/or biochemical HA, (ii) OD, and (iii) PCOM [2, 7]. PMOS is characterized by marked heterogeneity and is categorized into four phenotypes based on the Rotterdam criteria: “Phenotype A” includes HA, OD, and PCOM; “Phenotype B,” HA and OD; “Phenotype C,” HA and PCOM; and “Phenotype D,” OD and PCOM [8].

Metabolic abnormalities, such as insulin resistance, obesity, and type 2 diabetes mellitus, and psychological disorders, such as anxiety, depression, and eating disorders, are common comorbidities in PMOS. Furthermore, even after adjusting for obesity and other potential confounding factors, PMOS confers an increased risk of pregnancy complications, including gestational diabetes mellitus [9, 10].

Several racial and ethnic differences characterize PMOS phenotypes and its associated metabolic features [2, 11]. The manifestational degree of biochemical and clinical HA, obesity, insulin resistance, and psychiatric and cardiovascular risks exhibits a marked variation across populations. Phenotypes A and B predominate in Western, Mediterranean, and Middle Eastern populations and are frequently associated with high body mass index (BMI), overt obesity, and increased cardiometabolic risk. Phenotypes C and D are more common in East Asian populations and are generally associated with a low overall BMI, albeit increased central adiposity and insulin resistance, indicating metabolic risk despite a relatively lean phenotype and milder clinical HA, including less severe hirsutism [12, 13].

Although PMOS pathophysiology remains incompletely understood, HA is well‐recognized as a key contributor to disease development via complex interactions with OD, gonadotropin secretion abnormalities, hypothalamic–pituitary‐ovarian (HPO) axis dysregulation, and metabolic dysfunction. In particular, HA and insulin resistance interact bidirectionally, forming a vicious cycle that promotes PMOS development and progression. High‐frequency pulses of gonadotropin‐releasing hormone (GnRH) lead to excessive luteinizing hormone (LH) release, triggering hypersecretion of androgen by ovarian theca cells. A concurrent relative reduction in follicle‐stimulating hormone (FSH) impairs follicular maturation, causing the accumulation of preantral and small antral follicles and hypersecretion of anti‐Müllerian hormone (AMH), which, in turn, suppresses aromatase expression and further enhances GnRH/LH secretion, reinforcing HA. Additionally, hyperinsulinemia secondary to insulin resistance enhances ovarian androgen production and suppresses hepatic sex hormone–binding globulin (SHBG) synthesis, thereby increasing circulating free androgen levels. Obesity and visceral adiposity further amplify these endocrine abnormalities, contributing to the diverse clinical phenotypes of PMOS [14, 15].

### 2.2 Etiology of PMOS

Familial clustering and twin studies have demonstrated the high inheritability of PMOS. A Swedish nationwide registry‐based study reported that daughters of women with PMOS have a five‐fold increased risk of developing the condition [16]. A case–control study reported that daughters of women with PMOS exhibited higher LH, androgen, and insulin levels during the postmenarcheal period than controls, suggesting an increased risk of developing PMOS later in life [17]. In a Netherlands‐based large population‐based twin cohort study, higher concordance rates were observed in monozygotic twins (71%) than in dizygotic twins (38%), supporting a substantial genetic contribution to PMOS [18].

Genome‐wide association studies (GWAS) have advanced the understanding of the genetic architecture of PMOS while highlighting the complexity of its pathogenesis. Notably, evidence indicates a shared genetic architecture across PMOS phenotypes, with limited evidence for phenotype‐specific genetic differences, suggesting that genetic factors alone are insufficient to fully explain the phenotypic heterogeneity [19, 20, 21, 22]. GWAS have substantially expanded the number of PMOS susceptibility loci, supporting the concept of PMOS as a polygenic disorder: from 30 loci in earlier cross‐ancestry studies to > 90 independent loci in the recent multi‐ancestry analyses. These loci include genes involved in gonadotropin regulation (*FSHB* [FSH B subunit], *LHCGR* [LH/choriogonadotropin receptor]), ovarian function (*DENND1A* [DENN domain containing 1A]), and metabolic pathways (*INSR* [insulin receptor] and *THADA* [thyroid adenoma‐associated]).

Importantly, currently identified genetic susceptibility loci account for only a small proportion of its estimated heritability, a phenomenon referred to as “missing heritability.” This discrepancy may be attributable to the limited ability of GWAS to detect rare variants, and the influence of environmental and epigenetic factors. Although whole‐genome sequencing has identified rare variants in ovarian function and AMH signaling genes, these represent only a small proportion of disease susceptibility genes [23, 24, 25].

**FIGURE 1:**
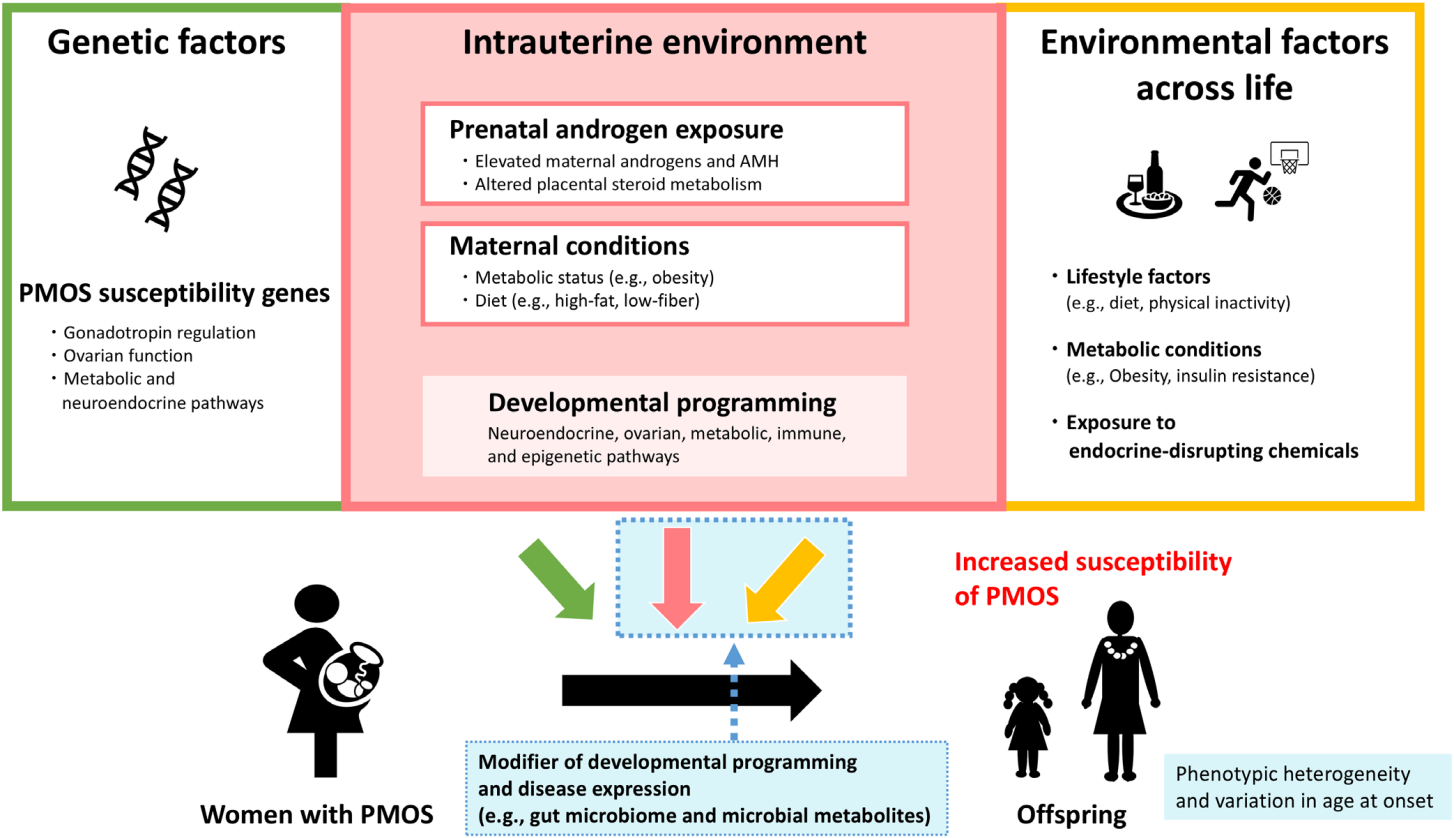
Etiology of PMOS: Integration of genetic susceptibility, developmental programming, and environmental factors. PMOS, a highly heritable disease, is characterized by complex interactions between genetic susceptibility and environmental influences. Prenatal androgen exposure, potentially driven by maternal hyperandrogenism and altered placental function, is a key developmental factor that may influence multiple developmental pathways during critical windows, including neuroendocrine, ovarian, metabolic, immune, and epigenetic programming, thereby contributing to long‐term alterations in reproductive and metabolic function. In addition, maternal conditions, including metabolic status and diet, and other intrauterine environmental factors may further shape developmental programming. Throughout life, additional environmental factors, including lifestyle, metabolic conditions, and endocrine‐disrupting chemicals, may further modulate disease susceptibility and phenotypic expression. Although these factors contribute to PMOS development, they do not fully explain its marked clinical heterogeneity or variability in disease onset. The gut microbiome may represent an additional environmental modifier that interacts with host developmental programming and genetic susceptibility. PMOS, polyendocrine metabolic ovarian syndrome.

Environmental influences such as early‐life exposures (prenatal androgen excess and maternal metabolic conditions), lifestyle factors (obesity and physical inactivity), and endocrine‐disrupting chemicals could interact with genetic susceptibility. Among these, prenatal androgen exposure is widely recognized as a key programming factor for the offspring's reproductive and metabolic dysfunction later in life [23, 26, 27]. Collectively, PMOS pathogenesis reflects a complex interplay of polygenic, epigenetic, and developmental factors, modulated by environmental influences (Figure 1).

### 2.3 Prenatal Androgen Exposure

Evidence confirms that daughters of women with PMOS are exposed to elevated androgen levels in utero. From a maternal perspective, pregnant women with PMOS exhibit higher circulating androgen levels than those without PMOS [28]. Furthermore, reduced placental P450 aromatase activity in women with PMOS may further contribute to a hyperandrogenic intrauterine environment [29]. From an offspring perspective, daughters of women with PMOS exhibit a longer anogenital distance (AGD), a well‐established biomarker of excessive intrauterine androgen exposure during both fetal and neonatal periods [30, 31].

Animal models, including rodents, sheep, and rhesus monkeys, provide compelling evidence supporting the causal role of prenatal androgen exposure in PMOS development later in life. Across multiple species, prenatal androgen exposure induced reproductive and metabolic phenotypes in adulthood that closely resembled those observed in women with PMOS [32, 33]. Prenatally androgenized (PNA) models are produced by exposing pregnant animals to dihydrotestosterone (DHT), or other non‐aromatizable androgens [24]. The timing of androgen susceptibility varies considerably across species, reflecting differences in placental maturation, hypothalamic–pituitary axis development, and gonadal differentiation. In rodents, androgen susceptibility occurs during mid‐to‐late gestation and the early postnatal period, whereas in primates it occurs mid‐gestation, when neuroendocrine and ovarian programming converge [27, 34]. These interspecies differences should be considered when extrapolating findings from PNA models to humans.

The molecular mechanisms via which prenatal androgen exposure contributes to PMOS development remain partially understood. However, PNA model studies indicate the central role of androgen receptor (AR)‐mediated signaling. In AR knockout mice, PNA‐induced PMOS‐like phenotypes are largely absent, suggesting that fetal programming effects are predominantly AR‐dependent [35, 36]. These findings indicate that AR signaling is a major mediator of the developmental effects of prenatal androgen exposure.

Accumulating evidence indicates that prenatal androgen exposure exerts organizational effects during critical windows of development across multiple organs and regulatory systems, including the neuroendocrine axis, ovary, adipose tissue, immune system, and epigenome. These parallel developmental pathways likely interact to determine long‐term reproductive and metabolic phenotypes.

Among these pathways, neuroendocrine developmental programming is considered a central mechanism underlying PMOS pathogenesis. Prenatal androgen exposure is thought to induce long‐lasting changes in the development of the HPO axis, thereby contributing to the neuroendocrine abnormalities characteristic of PMOS. Studies in PNA animal models have consistently demonstrated increased GnRH neuronal activity, impaired progesterone negative feedback, and persistently elevated LH pulsatility. Altered activity of upstream neuronal populations, including γ‐aminobutyric acid (GABA)‐ergic and kisspeptin neurons, has also been implicated in this process [37, 38]. AMH has recently emerged as an additional regulator of fetal neuroendocrine programming. Maternal AMH levels are elevated during pregnancy in women with PMOS and may suppress placental aromatase (CYP19A1) expression, thereby reducing the conversion of maternal androgens to estrogens and potentially increasing fetal androgen exposure. Moreover, AMH has been shown to directly influence fetal neuroendocrine development via GnRH neuronal activity modulation, suggesting that it may contribute to neuroendocrine programming through both androgen‐dependent and androgen‐independent mechanisms [35, 39, 40, 41].

Prenatal androgen exposure is also thought to directly influence ovarian development, resulting in long‐lasting alterations in folliculogenesis and steroidogenesis. Studies in PNA animal models have demonstrated altered folliculogenesis and granulosa‐ and theca‐cell function, with enhanced theca‐cell androgenic capacity preceding the development of overt PCOM [32, 42, 43]. These findings suggest that prenatal androgen exposure induces intrinsic ovarian developmental programming, rendering the ovary more susceptible to reproductive dysfunction later in life.

Prenatal androgen exposure has also been associated with persistent metabolic alterations. Studies in PNA animal models have demonstrated increased visceral adiposity, adipocyte hypertrophy, and insulin resistance following prenatal androgen exposure [44, 45]. Emerging evidence suggests that these metabolic abnormalities are not merely secondary to reproductive dysfunction but may reflect parallel developmental programming of metabolic tissues. Emerging evidence suggests that prenatal androgen exposure may contribute to persistent immune dysregulation and chronic low‐grade inflammation, although the mechanisms underlying immune developmental programming remain incompletely understood [32].

Epigenetic alterations have also been implicated in the long‐term effects of prenatal androgen exposure [3]. Animal studies have demonstrated persistent changes in DNA methylation following prenatal androgen or AMH exposure, including DNA methylation alterations in visceral adipose tissue of PNA rhesus monkeys and ovarian DNA hypomethylation in prenatal AMH‐exposed mice, the latter of which was partially reversed by methyl donor supplementation [46, 47]. In women with PMOS, differential DNA methylation has been identified in granulosa cells, adipose tissue, and peripheral blood, particularly in genes involved in steroidogenesis, insulin resistance, inflammatory response, and folliculogenesis [48, 49, 50]. Related epigenetic alterations have also been identified in genomic DNA extracted from peripheral blood leukocytes of infants aged 2–3 months born to women with PMOS, particularly in genes involved in androgen signaling and metabolic regulation [51]. Collectively, these findings suggest that epigenetic regulation may contribute to the persistent effects of prenatal androgen exposure. However, whether these epigenetic alterations represent primary drivers of developmental programming, downstream consequences of other programmed processes, or adaptive responses during disease progression remains to be determined.

The effects of prenatal androgen exposure are not limited to female offspring. Male offspring of women with PMOS exhibit potential risks for adverse metabolic and neurodevelopmental outcomes, albeit with less consistency than female offspring. Human studies suggest increased risks of insulin resistance, dyslipidemia, central adiposity, and neurodevelopmental disorders such as attention‐deficit/hyperactivity disorder and autism spectrum disorder. However, reproductive endocrine abnormalities appear less pronounced in male offspring. PNA animal models further support these observations, demonstrating metabolic dysfunction, behavioral alterations, and impaired reproductive parameters, in some cases, in male offspring. Overall, these findings suggest that prenatal androgen exposure influences long‐term health in male offspring as well, although variability across studies necessitates further investigation [52, 53, 54, 55, 56].

Environmental factors throughout life may further modify these prenatally programmed phenotypes. Experimental studies combining prenatal androgen exposure with a high‐fat diet (HFD) have demonstrated more pronounced reproductive and metabolic abnormalities than either intervention alone [57]. These findings further suggest that environmental exposures across the life course may modify the developmental effects of prenatal androgen exposure.

In summary, accumulating evidence suggests that prenatal androgen exposure influences multiple, interconnected developmental programs involving neuroendocrine, ovarian, metabolic, immune, and epigenetic pathways. These pathways likely interact to determine long‐term PMOS susceptibility but do not fully explain its phenotypic heterogeneity or clinical variability. These findings suggest that developmental programming may interact with additional modifiers, including the gut microbiome and postnatal environmental factors, to influence long‐term susceptibility to PMOS.

## 3 Gut Microbiome

The human gut harbors > 1,000 species and > 100 trillion microorganisms that form a complex host‐microbial community. *Microbiota* refers to the community of living microorganisms, whereas *microbiome* encompasses the microorganisms and their collective genetic and functional capacity [58]. With an estimated 3.3 million unique genes, approximately 150‐fold the size of the human genome, the gut microbiome influences a wide range of physiological processes via interactions with the nervous, endocrine, immune, and metabolic systems [59, 60, 61].

Gut microbiota composition is strongly shaped by dietary patterns and living environment, and has marked regional and ethnic variations. Western urban populations exhibit a *Bacteroides*‐dominant pattern, reflecting an animal protein and fat‐rich diet. Conversely, South and Southeast Asian populations often exhibit a *Prevotella*‐dominant pattern, reflecting a high carbohydrate and dietary fiber‐rich diet [62, 63, 64]. Japanese populations, whose traditional diet includes fermented foods and seaweed, harbor a gut microbiota enriched in Bifidobacterium, and certain Bacteroides strains possess the capacity to degrade seaweed‐derived polysaccharides, reflecting functional adaptation to long‐term dietary habits. These taxonomic and functional features together define a distinctive microbiome profile in Japanese populations [65, 66, 67]. However, such population‐specific microbiome features are dynamic and may shift with urbanization and dietary changes [68, 69].

**FIGURE 2:**
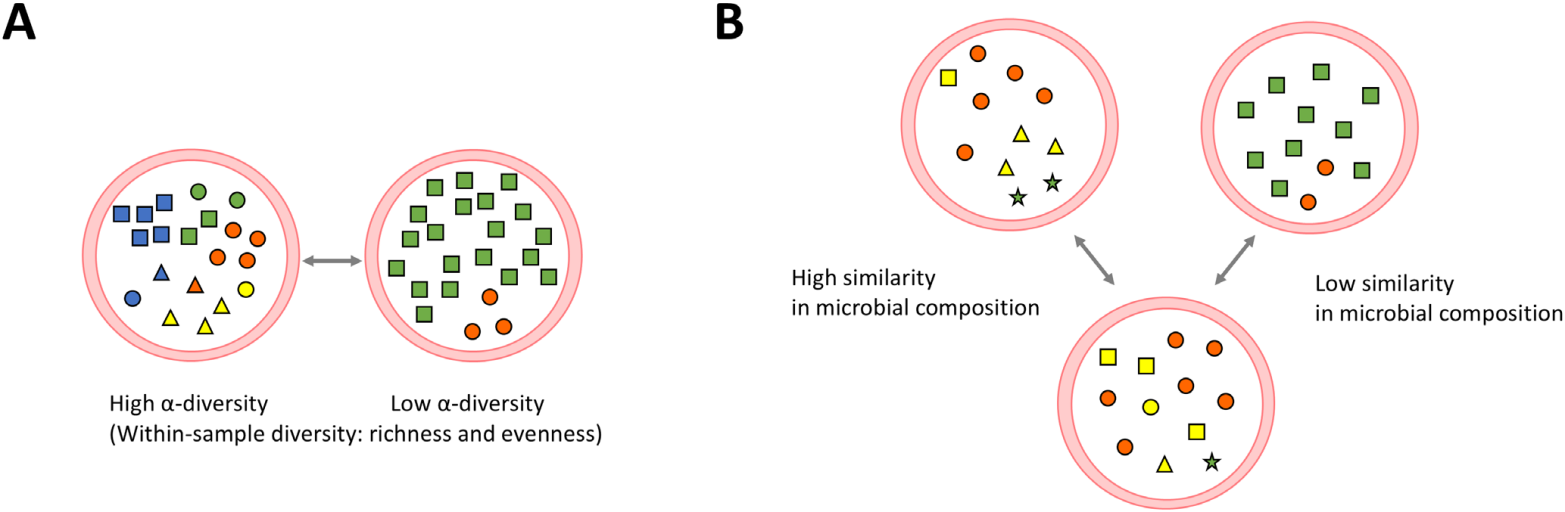
Conceptual illustration of α‐ and β‐diversity in the gut microbiota. (A) α‐diversity represents the diversity within a single sample, reflecting both richness (number of taxa) and evenness (distribution of taxa). Higher α‐diversity indicates a more diverse microbial community. (B) β‐diversity represents differences in microbial community composition between samples, reflecting variation in taxonomic composition and relative abundance.

Current gut microbiome studies involve DNA‐based analyses of fecal samples, including 16S rRNA and shotgun metagenomic sequencing techniques, thus enabling a comprehensive assessment of microbial composition and functional potential without the need for bacterial culture [70, 71]. Gut microbiota characteristics are evaluated in terms of diversity as well: α‐diversity represents microbial richness and evenness within a single sample, whereas β‐diversity reflects inter‐individual or inter‐group differences in microbial composition [72]. Community composition is assessed using relative abundance analysis, which indicates the proportion of each taxon within the overall community, and differential abundance analysis (via linear discriminant analysis effect size), which identifies bacterial taxa that significantly increase or decrease between groups (Figure 2). Regarding functional analysis, the Phylogenetic Investigation of Communities by Reconstruction of Unobserved States (PICRUSt) software predicts metabolic pathways from 16S rRNA data on reference genomes, whereas shotgun metagenomics directly identifies the genes and pathways present in microbial DNA. Importantly, microbiome data are influenced by sample collection and storage procedures, analytical methods, and biological factors such as sampling timing, thus requiring cautious interpretation of the results [73, 74].

Advanced analytical approaches have strongly evidenced the critical role of the gut microbiome in disease development. The gut microbiome is increasingly recognized as both a consequence of disease and as an active pathogenesis contributor in inflammatory bowel disease and metabolic disorders such as obesity and type 2 diabetes. Gut microbiota transplantation from individuals with obesity into germ‐free mice reportedly induced increased adiposity and insulin resistance without food intake changes, providing direct evidence of causality [75]. Mechanistic studies have further elucidated the pathways linking specific microbial species, their metabolites, host signaling, and downstream disease phenotypes [76]. Microbial metabolites can regulate histone modifications and DNA methylation, epigenetically modulating immune, metabolic, and endocrine systems [77, 78]. These mechanisms provide a potential biological link between early‐life environmental exposures and long‐term developmental programming.

Collectively, these findings suggest that the gut microbiome may act as a modifier linking developmental programming with PMOS susceptibility. Through its interactions with metabolic, endocrine, immune, and epigenetic pathways, the gut microbiome may contribute to phenotypic heterogeneity as well as disease onset and progression.

## 4 PMOS and the Gut Microbiome

### 4.1 Gut Microbiota Alterations in Women With PMOS and in Animal Models

The relationship between PMOS and the gut microbiome has received increasing research attention. Tremellen and Pearce's 2012 “dysbiosis of gut microbiota (DOGMA)” hypothesis is one among the earliest conceptual frameworks to link gut microbiota to PMOS [79]. It proposes that diet‐induced gut microbiota dysbiosis increases intestinal permeability (“leaky gut”), leading to chronic low‐grade inflammation mediated by lipopolysaccharides (LPS) derived from Gram‐negative bacteria. This inflammatory state may promote insulin resistance, contributing to HA, with bidirectional interaction of these processes driving PMOS pathogenesis.

Experimental evidence from letrozole‐induced PMOS rodent models demonstrated that gut microbiota composition differed between PMOS models and healthy controls [80, 81]. Notably, the letrozole‐induced PMOS‐like phenotypes differed depending on the timing of letrozole administration: reproductive abnormalities were observed in the pubertal or adulthood models, whereas metabolic abnormalities and obesity‐associated gut dysbiosis were predominantly observed in the pubertal model. Thus, the metabolic and microbial features of PMOS may depend on the developmental timing of androgen exposure [82]. Similarly, prenatal or neonatal androgen exposure‐induced PMOS models have consistently demonstrated alterations in gut microbiota composition in adulthood [83, 84, 85]. Subsequent human observational studies, conducted across diverse ethnic populations, have reported that women with PMOS exhibit distinct gut microbiota profiles compared with healthy controls, consistent with findings from animal studies [26, 86, 87, 88]. Several recent reviews have reported reduced α‐diversity and distinct community structures in women with PMOS, although the results are not entirely consistent. Furthermore, associations between specific microbial taxa and clinical parameters, including circulating androgen levels and metabolic phenotypes, have been observed [89, 90, 91]. Although accumulating studies have reported gut microbiota dysbiosis in adult women with PMOS, relatively few studies have investigated gut microbiota alterations in childhood or adolescence. Nevertheless, reduced α‐diversity and changes in β‐diversity have been reported in adolescents with PMOS even after accounting for obesity, suggesting that gut microbiota alterations may already be present early in the disease course [92, 93].

Across studies, the specific bacterial taxa reported as increased or decreased vary depending on species, experimental models, and analytical methodologies; however, gut microbiota alterations in PMOS have consistently been observed [89], suggesting the overall structure and functional capacity of the gut microbiome, particularly its metabolite profiles, may be more relevant to PMOS pathogenesis than individual taxa. Importantly, functional analyses of the gut microbiome in PMOS have identified alterations in bile acid metabolism, short‐chain fatty acid (SCFA) production, inflammatory pathways, and steroid deconjugation capacity [94, 95].

Despite these associations, whether these alterations are causal or secondary to the disease remains unclear. In particular, metabolic disorders represent major confounders affecting both gut microbiota composition and PMOS, complicating causal inference. Some animal model studies have reported that FMT or co‐housing interventions that modify gut microbiota improve PMOS‐like features, including estrous cyclicity, circulating androgen levels, and body composition [81, 96]. However, others have reported no improvement in PMOS‐like phenotypes after microbiota manipulation [97, 98], suggesting that these discrepancies may reflect differences in experimental models, timing of intervention, or methodological factors. Clinical studies have reported that probiotics, synbiotics, and dietary interventions improve certain reproductive and metabolic parameters in women with PMOS; nevertheless, substantial heterogeneity in study design, including differences in bacterial strains, dosage, and treatment duration, limits definitive conclusions regarding clinical efficacy [99, 100]. Although Mendelian randomization analyses have suggested potential causal associations between certain gut microbial taxa and PMOS, the available evidence remains limited by methodological constraints and requires further validation [101, 102].

### 4.2 Gut Microbiome‐Mediated Pathophysiological Mechanisms of PMOS

**FIGURE 3:**
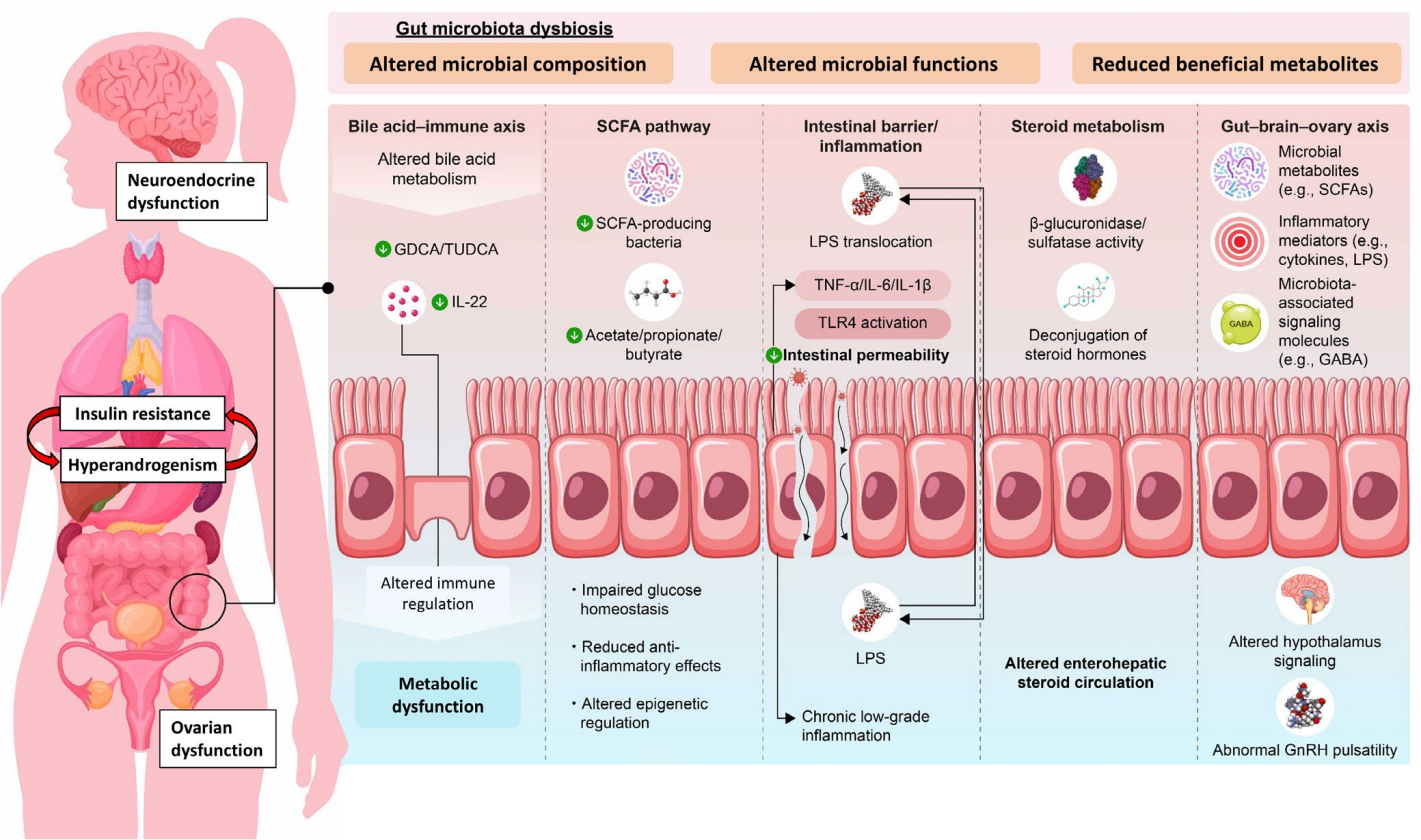
Functional mechanisms linking the gut microbiome to PMOS pathophysiology. Functional alterations of the gut microbiome may contribute to PMOS pathophysiology via multiple interconnected pathways, including bile acid metabolism, short‐chain fatty acid and tryptophan metabolism, steroid metabolism, intestinal barrier dysfunction, immune dysregulation, and neuroendocrine signaling. These pathways may influence insulin resistance, hyperandrogenism, chronic inflammation, and ovarian dysfunction. Bidirectional interactions between hyperandrogenism and gut dysbiosis further exacerbate disease progression. GABA, γ‐aminobutyric acid; GDCA, glycodeoxycholic acid; IL‐22, interleukin‐22; LPS, lipopolysaccharide; PMOS, polyendocrine metabolic ovarian syndrome; SCFA, short‐chain fatty acid; TLR4, Toll‐like receptor 4; TNF‐α, tumor necrosis factor‐α; TUDCA, tauroursodeoxycholic acid.

Although taxonomic alterations vary considerably among studies and ethnic populations, increasing attention has shifted toward functional characterization of the gut microbiome to better understand the biological mechanisms underlying PMOS. Functional pathways involving microbial metabolites, steroid metabolism, immune regulation, intestinal barrier function, and neuroendocrine signaling have emerged as key areas of investigation (Figure 3).

Altered microbial bile acid metabolism has emerged as an important functional pathway linking the gut microbiome to immune regulation in PMOS. Women with PMOS exhibit an increased abundance of *Bacteroides vulgatus* , accompanied by decreased levels of bile acids glycodeoxycholic acid (GDCA) and tauroursodeoxycholic acid (TUDCA), together with decreased interleukin (IL)‐22 production. Functional studies further demonstrated that modulation of the bile acid–IL‐22 axis influences both metabolic and reproductive phenotypes, supporting a mechanistic role for gut microbiota‐derived bile acid metabolism in PMOS pathophysiology [103].

Among microbiota‐derived metabolites, SCFAs, particularly butyrate, may play an important role in PMOS pathophysiology. SCFAs regulate host physiology through multiple pathways, including free fatty acid receptors 2 and 3 (FFAR2 and FFAR3; formerly GPR43 and GPR41, respectively)–mediated signaling, maintenance of intestinal epithelial integrity, modulation of mucosal and systemic immune responses, and epigenetic regulation through histone deacetylase (HDAC) inhibition [77, 104]. These actions may influence insulin sensitivity, low‐grade inflammation, and endocrine homeostasis, all of which are key features of PMOS. Consistent with these findings, reduced abundance of SCFA‐producing bacteria and altered fecal SCFA profiles have been reported in women with PMOS and in animal models [84, 105, 106]. Furthermore, butyrate supplementation has ameliorated reproductive and metabolic dysfunction in animal models, providing functional evidence that impaired SCFA signaling may contribute to disease pathophysiology [107, 108].

Emerging evidence suggests that altered microbial tryptophan metabolism contributes to PMOS pathophysiology. Blood metabolomic studies in women with PMOS have demonstrated disturbances in tryptophan metabolism, including alterations in the host kynurenine pathway, which have been associated with insulin resistance, systemic inflammation, and reproductive endocrine abnormalities [109, 110]. Gut microbial tryptophan metabolites, such as indole‐3‐propionic acid (IPA), can regulate intestinal barrier function, immunity, and metabolism, at least partly through aryl hydrocarbon receptor (AhR) signaling [111]. Consistently, IPA supplementation ameliorated PMOS‐like phenotypes in a DHEA‐induced mouse model, supporting a functional role for microbial tryptophan metabolism in PMOS [112]. Collectively, these findings suggest that microbial tryptophan metabolism represents another functional pathway linking the gut microbiome to host metabolism, immune regulation, and PMOS pathophysiology.

Gut microbiome‐mediated steroid metabolism represents another potential mechanism linking the gut microbiome to PMOS. Emerging evidence suggests that HA has a bidirectional relationship with the gut microbiome. Gut microbial β‐glucuronidase and sulfatase activities may promote deconjugation of steroid conjugates, thereby facilitating enterohepatic recirculation of sex steroids [113, 114, 115]. A mechanism analogous to that of the “estrobolome” in estrogen metabolism may exist for androgens [116]. However, direct evidence demonstrating that microbial reactivation of conjugated androgens determines circulating androgen levels or drives HA in PMOS remains limited. Conversely, accumulating evidence indicates that HA alters gut microbiota composition. Sex differences in gut microbiota, observed in both human and animal models, were partly reversible with manipulation of androgen levels by castration or androgen supplementation, suggesting that sex hormone‐dependent mechanisms shape microbial composition [114, 117, 118, 119]. In PMOS animal models, androgen administration or letrozole‐induced HA consistently resulted in reduced microbial diversity and shifts in specific bacterial taxa, suggesting a potential vicious cycle in PMOS, in which HA and gut dysbiosis mutually reinforce one another.

Gut microbiota dysbiosis may further contribute to PMOS by disrupting intestinal barrier integrity and promoting chronic low‐grade inflammation. Increased intestinal permeability may permit bacterial LPS translocation into the circulation, potentially activating Toll‐like receptor 4 (TLR4) signaling and increasing proinflammatory cytokine (tumor necrosis factor‐α, IL‐6, and IL‐1β) production. This inflammatory state may contribute to insulin resistance and compensatory hyperinsulinemia, which, in turn, enhances ovarian androgen production [120, 121]. Consistent with this concept, markers of impaired intestinal barrier function have been associated with metabolic and reproductive abnormalities in women with PMOS [88].

Additionally, the gut microbiome may influence the HPO axis via neuroendocrine pathways. Microbial metabolites, including SCFAs, together with inflammatory signals, may modulate hypothalamic function via the gut‐brain axis, potentially influencing GnRH pulsatility and downstream LH and FSH secretion [122, 123]. Consistent with this concept, sodium butyrate supplementation alleviated hypothalamic inflammation and partially restored hypothalamic GABAergic signaling in a letrozole‐induced PMOS rat model, supporting a role for gut microbiota‐derived SCFAs in neuroendocrine regulation [124].

Collectively, these findings suggest that functional alterations of the gut microbiome—including microbial metabolite production, steroid metabolism, immune regulation, intestinal barrier function, and neuroendocrine signaling—may contribute to the metabolic, endocrine, and immune dysfunction associated with PMOS.

### 4.3 Prenatal Androgen Exposure‐Induced Alterations in Gut Microbiota From Early Life and PMOS Development

**FIGURE 4:**
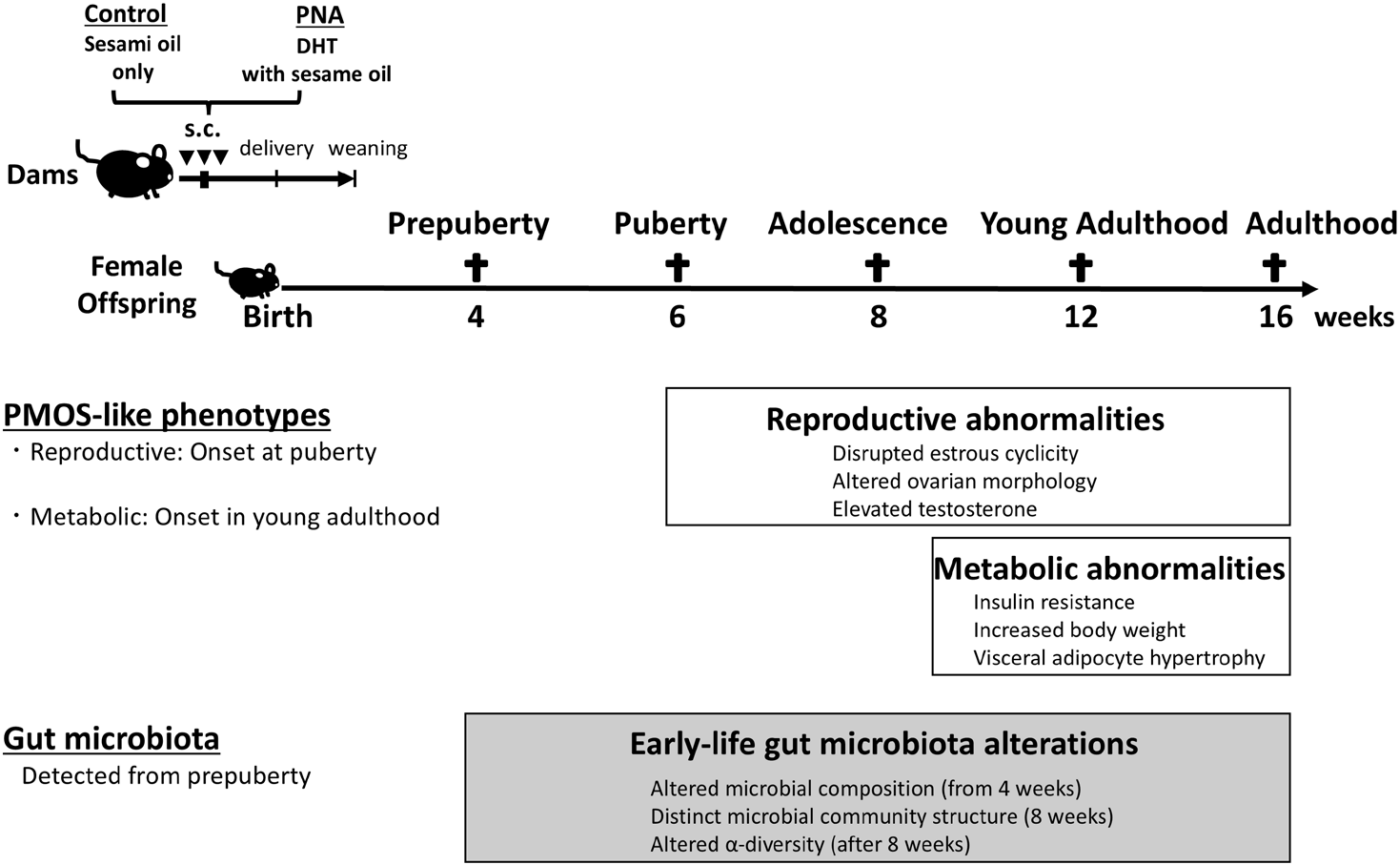
Temporal relationship between PMOS‐like phenotypes and alterations in the gut microbiota in PNA offspring. A PNA model was generated by subcutaneously (s.c.) injecting pregnant C57BL/6 mice with DHT on gestational days 16–18. PMOS‐like phenotypes and gut microbiota were compared between the PNA and control groups at 4 (prepuberty), 6 (puberty), 8 (adolescence), 12 (young adulthood), and 16 (adulthood) weeks (*n* = 12 each group). Reproductive abnormalities became apparent from puberty, whereas metabolic abnormalities developed during young adulthood. In contrast, alterations in gut microbiota composition were already detectable by prepuberty and persisted into adulthood. These findings demonstrate a temporal association between early‐life gut microbiota alterations and subsequent PMOS‐like phenotypes, suggesting that gut microbiota dysbiosis may represent one component of the developmental programming process. DHT, dihydrotestosterone; PMOS, polyendocrine metabolic ovarian syndrome; PNA, prenatally androgenized.

Based on current evidence linking prenatal androgen exposure, the gut microbiome, and PMOS pathophysiology, we hypothesized that excessive androgen exposure during the fetal period disrupts early‐life gut microbiota development and may contribute to PMOS development later in life. In our previous longitudinal study using a PNA mouse model, we characterized gut microbiota changes together with reproductive and metabolic phenotypes from weaning to adulthood (Figure 4) [125]. Female offspring were found to exhibit the reproductive features of PMOS at puberty, whereas metabolic abnormalities became apparent during young adulthood. In contrast, alterations in gut microbiota composition were already detectable during the prepubertal period and persisted thereafter, preceding the onset of overt metabolic abnormalities. These findings suggest that early‐life gut microbiota alterations are temporally associated with subsequent PMOS‐like phenotypes.

**FIGURE 5:**
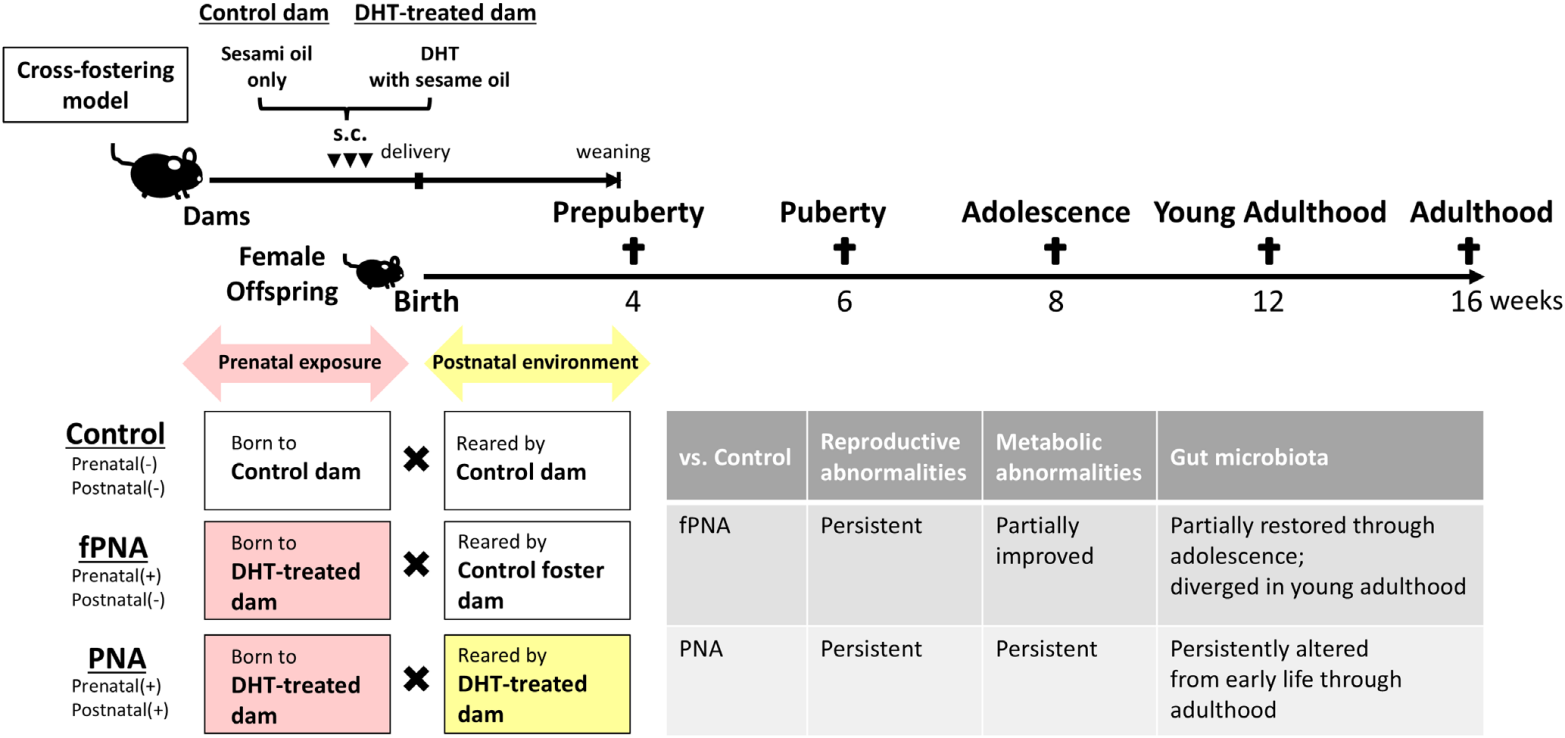
Relative contributions of prenatal androgen exposure and the postnatal environment to gut microbiome development and PMOS‐like phenotypes in a cross‐fostering mouse model. A cross‐fostering model was used to distinguish the relative contributions of prenatal androgen exposure and the postnatal environment to gut microbiota development and PMOS‐like phenotypes. Female offspring were assigned to three groups according to prenatal exposure and postnatal rearing conditions: Control (prenatal (−)/postnatal (−)), fPNA (prenatal (+)/postnatal (−)), and PNA (prenatal (+)/postnatal (+)). The figure summarizes the longitudinal changes in reproductive and metabolic phenotypes together with gut microbiota maturation from prepuberty to adulthood. The schematic summarizes that concept that prenatal androgen exposure is the primary determinant of reproductive abnormalities, whereas the postnatal environment may partially modify gut microbiota maturation and metabolic phenotypes. DHT, dihydrotestosterone; fPNA, fostered PNA offspring; PMOS, polyendocrine metabolic ovarian syndrome; PNA, prenatally androgenized.

To distinguish the relative contributions of prenatal androgen exposure and the postnatal environment (maternal gut microbiota, lactation, and maternal care), we next employed a cross‐fostering model in which pups were exchanged between dams immediately after birth and reared by foster mothers until weaning, enabling the separation of prenatal and postnatal influences (Figure 5). Abnormal postnatal environment referred to rearing by dams exposed to DHT during pregnancy, whereas normal postnatal environment referred to rearing by untreated control dams [126]. Although prenatal androgen exposure remained the primary determinant of reproductive and metabolic phenotypes, a normal postnatal environment partially attenuated metabolic abnormalities and delayed divergence of gut microbiota dysbiosis compared with conventionally reared PNA offspring. These findings indicate that early postnatal environmental factors, including maternal microbiota, lactation, and maternal care, may modulate gut microbiota maturation and partially modify disease expression following prenatal androgen exposure.

Importantly, these observations do not necessarily indicate that alterations in the gut microbiota are the sole mediator of developmental programming. Rather, together with previous evidence, they suggest that prenatal androgen exposure concurrently influences multiple developmental pathways—including neuroendocrine, metabolic, immune, and epigenetic processes—as well as the gut microbiota establishment. In turn, the gut microbiota may interact with these programmed processes, potentially modulating their long‐term effects. Collectively, these findings suggest that prenatal and early postnatal environments jointly shape both host development and microbiota establishment. Although the precise causal relationships remain incompletely understood, the early‐life gut microbiota may represent an important modifier of developmental programming and warrant further investigation as a potential target for future preventive or therapeutic strategies in PMOS.

## 5 Maternal–Infant Microbial Transmission and the Impact of the Intrauterine Environment on Developmental Programming

Given the potential role of the gut microbiome in developmental programming, understanding how the infant gut microbiota is established and shaped by maternal and early‐life environmental factors is essential. Meconium contains a miniscule amount of microorganisms, and infant gut microbiota establishment commences immediately post‐birth. In the early postnatal period, as intestinal oxygen levels decrease, facultative anaerobic bacteria such as *Enterobacteriaceae*, *Staphylococcus*, and *Streptococcus* dominate as the initial colonizers. The initiation of breastfeeding leads to the predominance of *Bifidobacterium*, which significantly shapes the infant's gut ecosystem [127]. The gut microbiota subsequently develops in a staged manner, progressing through the developmental (3–14 months), transitional (15–30 months), and stable (31–46 months) phases, ultimately reaching an adult‐like composition at approximately 3 years [128]. *Bifidobacterium* and *Lactobacillus*, which are important for immune development, predominate during early life. With the introduction of solid foods, *Bacteroides* and *Firmicutes* increase in abundance, accompanied by an enhanced SCFA production.

**FIGURE 6:**
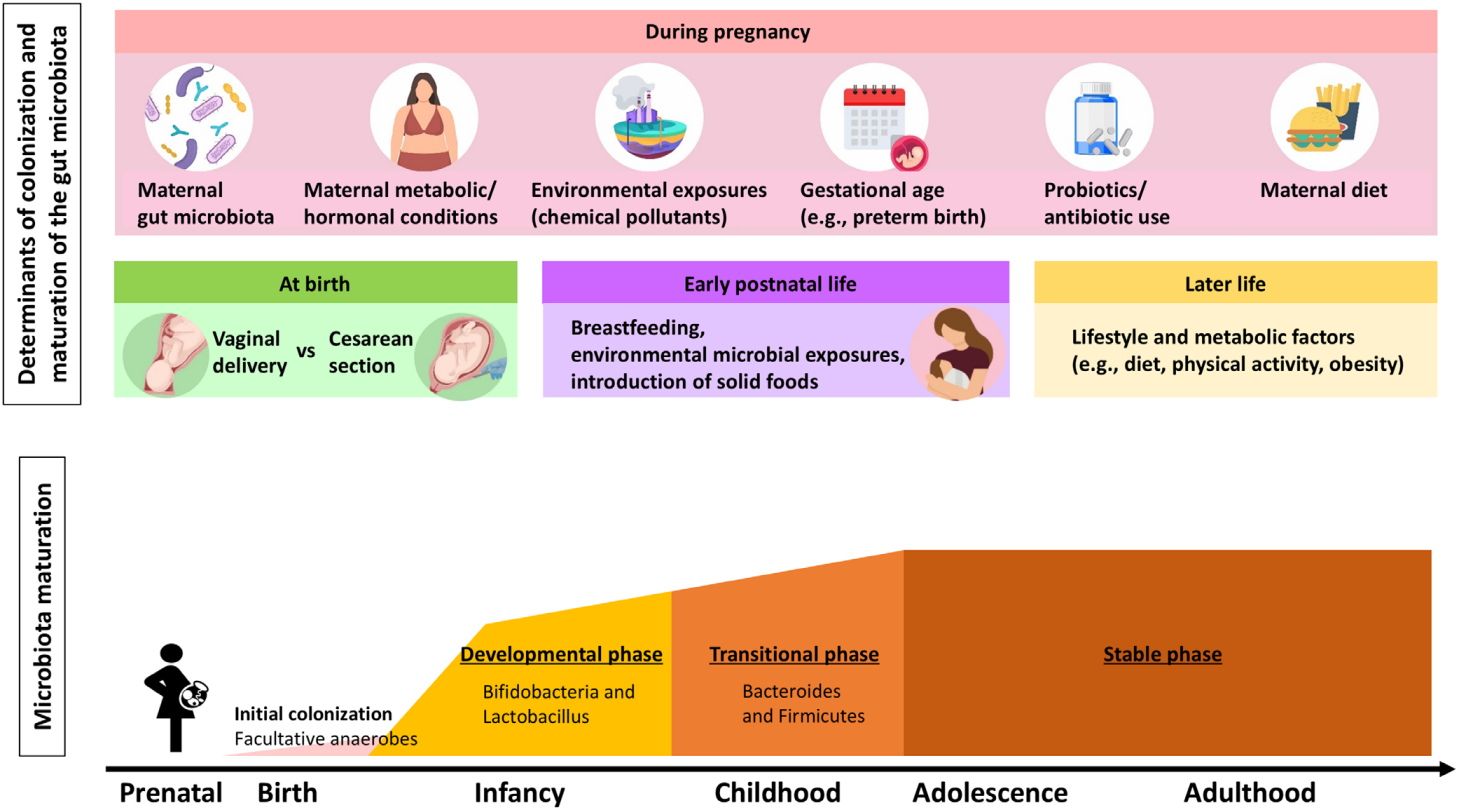
Maternal influences on early‐life microbiome establishment and developmental programming. Gut microbiota develops toward an adult‐like composition through defined developmental phases, with early life, particularly the first 3 years, representing a critical window for microbiome establishment. Maternal factors, including diet, metabolic and hormonal status, and microbiota composition, shape offspring gut microbiome development via prenatal, perinatal, and postnatal pathways. During pregnancy, maternal microbiota‐derived metabolites and hormonal signals may contribute to fetal programming. The mode of delivery is a major determinant of initial microbial colonization, whereas breastfeeding provides microbes and bioactive components, such as human milk oligosaccharides, that promote microbiome maturation. These processes influence immune, metabolic, and endocrine development, contributing to long‐term disease susceptibility, including PMOS. In the schematic, maternal factors are indicated in light red, perinatal factors (e.g., mode of delivery) in green, and postnatal/offspring‐related factors in light blue. These elements represent key determinants of colonization and maturation of the gut microbiota, highlighting the temporal and biological transitions that shape microbiome development. PMOS, polyendocrine metabolic ovarian syndrome.

Maternal factors play an important role in the early colonization and maturation of infant gut microbiota (Figure 6) [6, 127]. Delivery mode is one of the major determinants of initial microbial composition. During vaginal delivery, infants are initially exposed to maternal vaginal and fecal microbiota, whereas cesarean delivery alters this early microbial exposure. Vaginally delivered infants have an increased abundance of *Bacteroides, Bifidobacterium*, and *Escherichia* species, whereas cesarean‐delivered infants exhibit delayed colonization by these taxa and have an increased relative abundance of skin‐ and environment‐associated microbes during early life. Although these differences often attenuate over time, delivery mode can substantially influence early gut microbiota development [129, 130, 131]. Breastfeeding is another important route of maternal microbial and bioactive factor transfer, providing both microbes and human milk oligosaccharides, which selectively promote beneficial bacteria, as well as immune components and antimicrobial factors that shape microbiota development and host physiology [132, 133, 134, 135]. In addition, accumulating evidence suggests that maternal factors during pregnancy, including diet, metabolic status, and gut microbiota composition, influence early‐life microbiota composition and may influence early‐life gut microbiota composition and subsequent offspring health [136]. Infants born to mothers consuming a HFD exhibit distinct early‐life gut microbiota profiles compared with those born to mothers consuming a standard diet [137]. Consistent with these observations, animal studies demonstrate that maternal HFD induces early postnatal microbiota alterations in the offspring, which have been associated with behavioral changes [138].

The prenatal presence of microbiota has been suggested, although this remains debatable [139, 140]. Therefore, rather than direct microbial transfer, maternal influences on offspring gut microbiota development are considered to occur primarily via indirect mechanisms. Alterations in the maternal microbial environment modify the production of microbial metabolites, which can cross the placenta and influence fetal developmental programming, thereby shaping postnatal gut microbiota establishment. Among these metabolites, SCFAs have been most extensively studied. Maternal SCFAs are transferred to the fetus via the placenta and regulate embryonic energy metabolism through receptors such as GPR41 and GPR43, thereby affecting susceptibility to obesity and metabolic dysfunction later in life [141]. Consistent with this concept, offspring born to germ‐free mothers or mothers consuming a low‐fiber diet have an increased susceptibility to metabolic syndrome, whereas maternal SCFA supplementation during pregnancy mitigates these adverse outcomes. Moreover, maternal butyrate administration during pregnancy has been found to exert long‐term effects on offspring intestinal barrier function and inflammatory response [142]. These findings suggest that maternal microbial environments and microbial metabolites may contribute to offspring gut microbiome establishment and developmental programming.

## 6 Summary, Challenges, and Future Perspectives

This review has focused on how intrauterine and early postnatal environments shape developmental programming and summarized current evidence linking the gut microbiome to PMOS. Accumulating evidence suggests that the gut microbiome may modulate multiple aspects of PMOS pathophysiology, including HA, insulin resistance, chronic inflammation, and neuroendocrine dysfunction.

However, PMOS‐associated microbial signatures remain inconsistent across populations and experimental models, and no specific pathogenic bacteria have been definitively identified. Moreover, major host and environmental factors, such as obesity, insulin resistance, diet, and genetic background, substantially influence gut microbial composition, making it difficult to distinguish primary disease‐associated alterations from secondary changes. Although microbiome‐targeted interventions have shown promising results, heterogeneity in study design, analytical approaches, and intervention protocols limits reproducibility, thus highlighting the need for standardized approaches.

**FIGURE 7:**
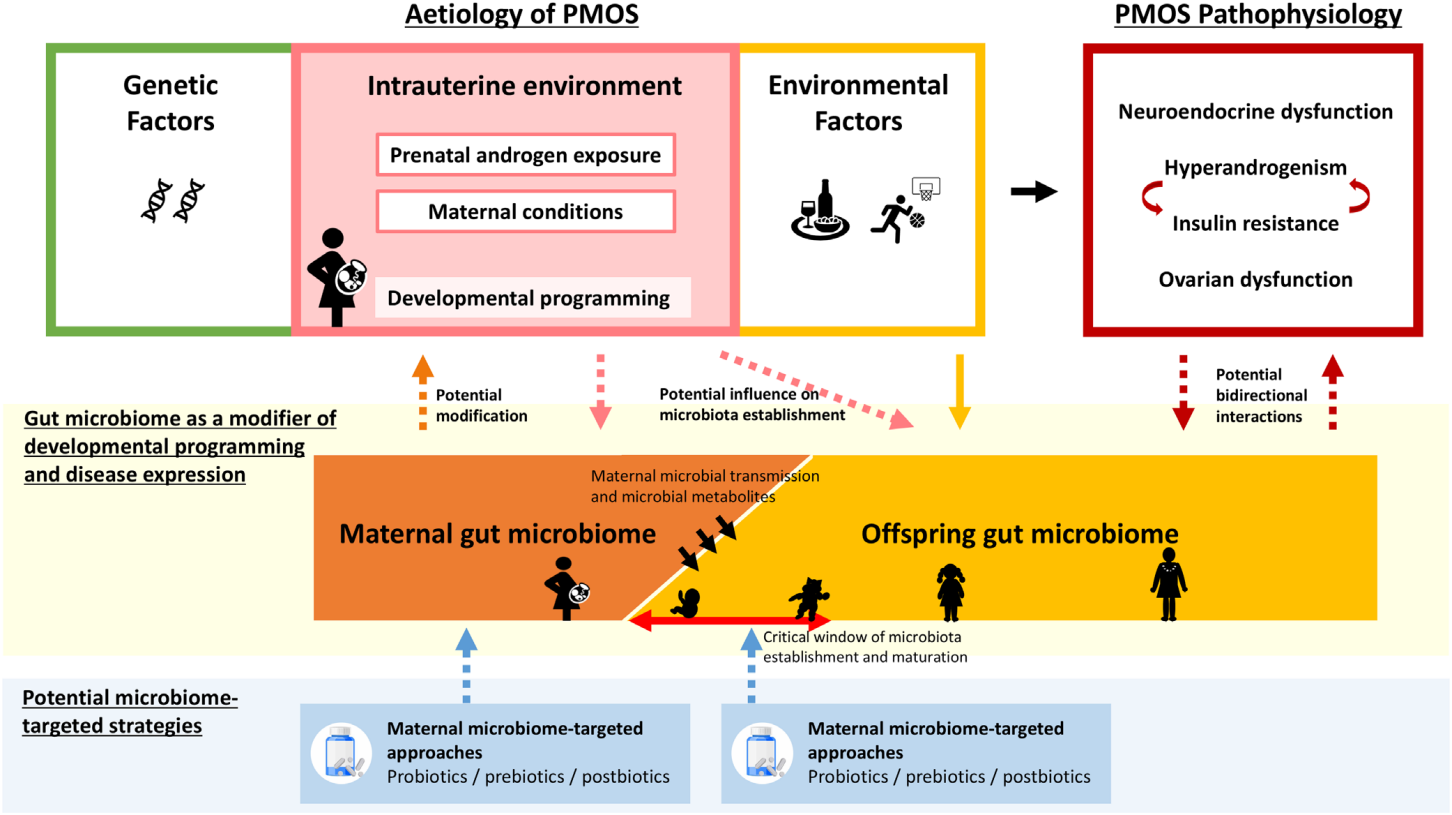
Conceptual framework of microbiome‐mediated developmental programming in PMOS and future directions. Genetic susceptibility, developmental programming, and environmental factors collectively contribute to PMOS pathogenesis. Prenatal androgen exposure and maternal conditions influence multiple developmental pathways during critical developmental windows, including neuroendocrine, ovarian, metabolic, immune, and epigenetic programming. Maternal microbial factors—including microbiota‐derived metabolites and maternal–offspring microbial transmission—together with early‐life microbiome establishment, may further modify host developmental programming and long‐term disease susceptibility. Throughout life, the gut microbiome may interact with multiple components of PMOS pathophysiology, including neuroendocrine and ovarian dysfunction, hyperandrogenism, and insulin resistance. Some of these relationships, particularly those involving hyperandrogenism and metabolic dysfunction, may be bidirectional, potentially contributing to disease expression and progression. Although causal relationships remain to be fully established, the gut microbiome may represent one component of developmental programming and a potential target for future preventive and therapeutic strategies, particularly during early life. Solid arrows indicate relationships supported by current evidence, whereas dashed arrows indicate proposed or hypothetical interactions requiring further investigation. PMOS, polyendocrine metabolic ovarian syndrome.

Importantly, emerging evidence suggests that disease susceptibility and phenotypic heterogeneity may depend more on the overall structure and functional capacity of the gut microbial ecosystem—including microbial metabolite and host microbiome interactions—than on individual bacterial taxa alone. Accordingly, microbiome research is transitioning from descriptive taxonomic profiling toward mechanistic and functional analyses. Future study should integrate multi‐omics approaches, including metagenomics, metabolomics, transcriptomics, epigenomics, and host genetic analyses, to identify functional microbial pathways and elucidate how genetic susceptibility interacts with prenatal and postnatal environmental exposures during critical developmental windows. Particular attention should be directed toward the prenatal and early postnatal periods, when developmental programming of both the host and the gut microbiome may exert long‐lasting effects on reproductive and metabolic health. As illustrated in Figure 7, the gut microbiome may represent an important modifier within a broader developmental programming network that interacts with neuroendocrine, metabolic, immune, and epigenetic pathways throughout life.

Despite the well‐recognized familial aggregation of PMOS, effective preventive strategies and fundamental treatments remain limited. Therefore, a deeper understanding of gut microbiome‐mediated PMOS pathogenesis may ultimately facilitate the development of microbiome‐targeted preventive strategies and personalized therapeutics.

## Funding

This work was supported by the Japan Society for the Promotion of Science, 26K02538, 25K20080, 23K19667, 22K0961419, Children and Families Agency, 26DA0401, 25DA0701.

## Conflicts of Interest

The authors declare no conflicts of interest.

## Data Availability

Data sharing not applicable to this article as no datasets were generated or analysed during the current study.
